# Supplementary material for: Aging drives cerebrovascular network remodeling and functional changes in the mouse brain
Source: Nat Commun. 2024 Jul 30;15:6398. doi: 10.1038/s41467-024-50559-8 (PMC11289283; doi:10.1038/s41467-024-50559-8)
Supplement: Supplementary file 1 — Supplementary Information [file 41467_2024_50559_MOESM1_ESM.pdf]

Supplementary Figure 1

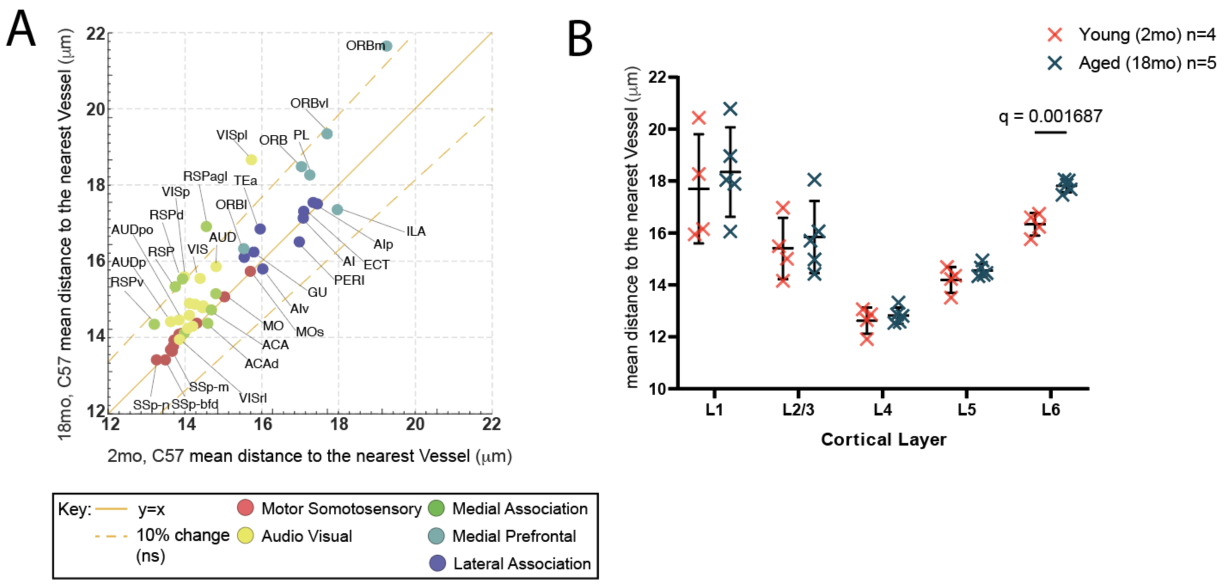

Nearest neighbor distance (NND) to vessels. (**A**) Scatter plots of averaged NND (μm) in isocortical areas. No areas show significant differences across 2mo (n=4) and 18mo (n=5). (**B**) NND showed significant reductions only in layer 6. Data are presented as mean values +/- SD. Brain region abbreviations can be found in Supplementary Data 1.

Supplementary Figure 2

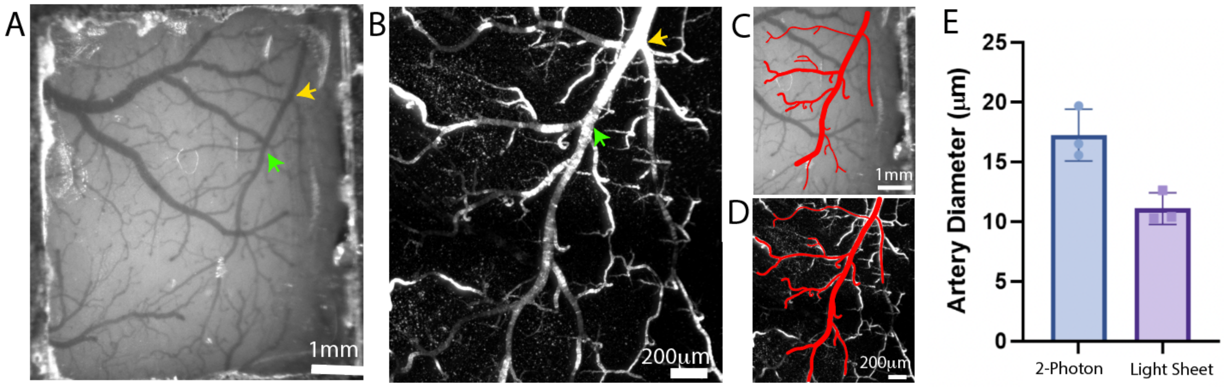

Intact vascular geometry after tissue clearing and immunolabeling. (**A-B**) In vivo two-photon imaging (**A**) and light sheet imaging (**B**) of the matched area from the same animal (n=3). (**C-D**) A selection of an artery (red) as an example in the two-photon imaging (**C**) and its overlay with scaling in the light sheet imaging (**D**). Note the near complete overlap. (**E**) Artery diameter measurement from the same artery shows ~36% decrease in the light sheet imaging after the tissue clearing. Scale bars for in vivo two-photon imaging are 1mm and scale bars for light sheet imaging are 200mm. Data are presented as mean values +/- SD.

Supplementary Figure 3

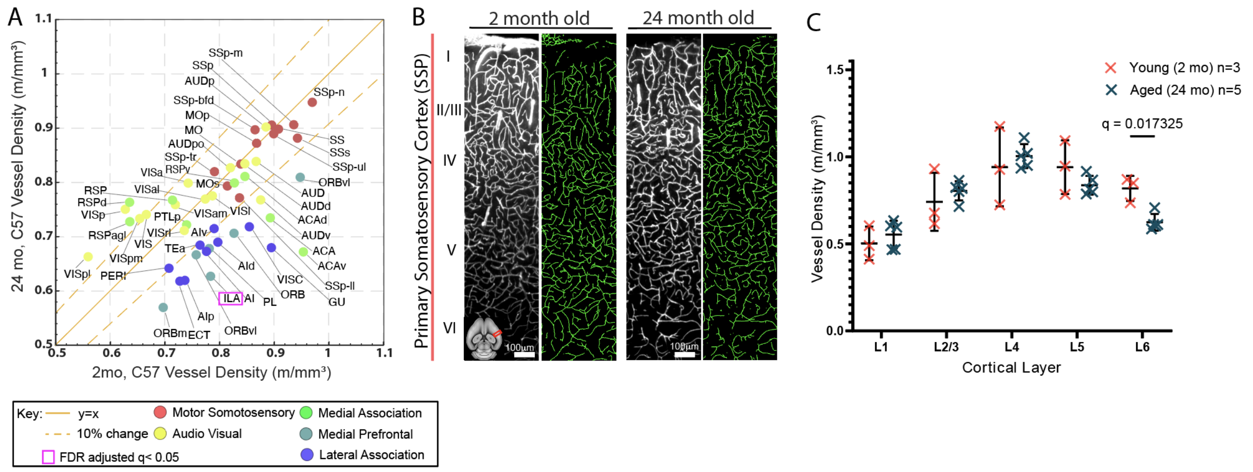

Selective reduction of vascular length density in the late aging mice. (**A**) Scatter plots of vascular length density (m/mm^3) in isocortical areas from 2-month-old (n=4) and 24-month-old mice (n=5) by light sheet imaging. Only the infralimbic cortex shows a significant difference. (**B**) Examples of light sheet imaging with lectin pan-vascular staining (left) and tracing (right, green) from 2-month-old (n=3) and 24-month-old mice (n=5). Scale bars are 100mm. (**C**) Vascular length density across cortical layers showed that only layer 6 showed significant reduction in the aged brain, when comparing 2-month-old (n=3) and 24-month-old mice (n=5). Data are presented as mean values +/- SD.

Supplementary Figure 4

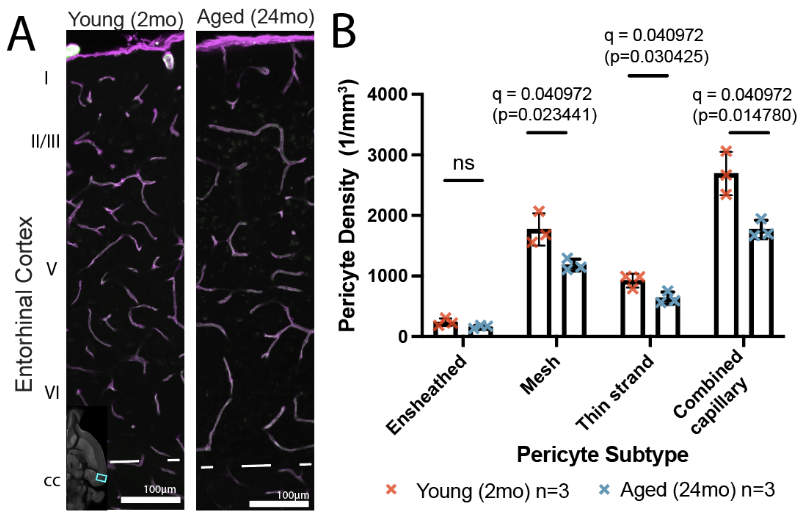

Significant reduction of pericytes in the entorhinal cortex. (**A-B**) The entorhinal cortex (**A**) showed a significant reduction of capillary pericytes (**B**), from 2-month-old (n=3) and 24-month-old mice (n=3). Scale bars are 100mm. Data are presented as mean values +/- SD for all graphs

Supplementary Figure 5

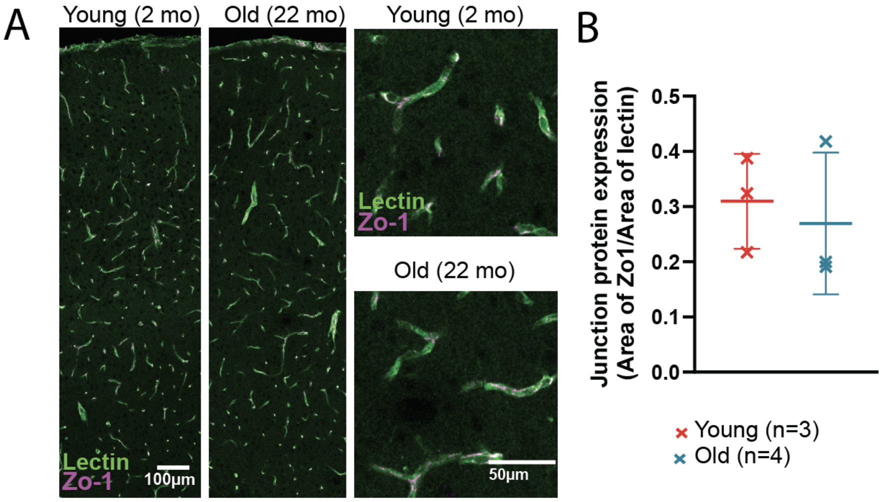

Lack of significant changes in the Zo-1 expression in the aged brain. (**A**) Zo-1 and Lectin staining in 2-month-old (n=3) and 22-month-old brains (n=4). (**B**) No significant difference between the two age groups. Scale bars for main images are 100µm and for high magnification images are 50µm. Data are presented as mean values +/- SD.

Supplementary Figure 6

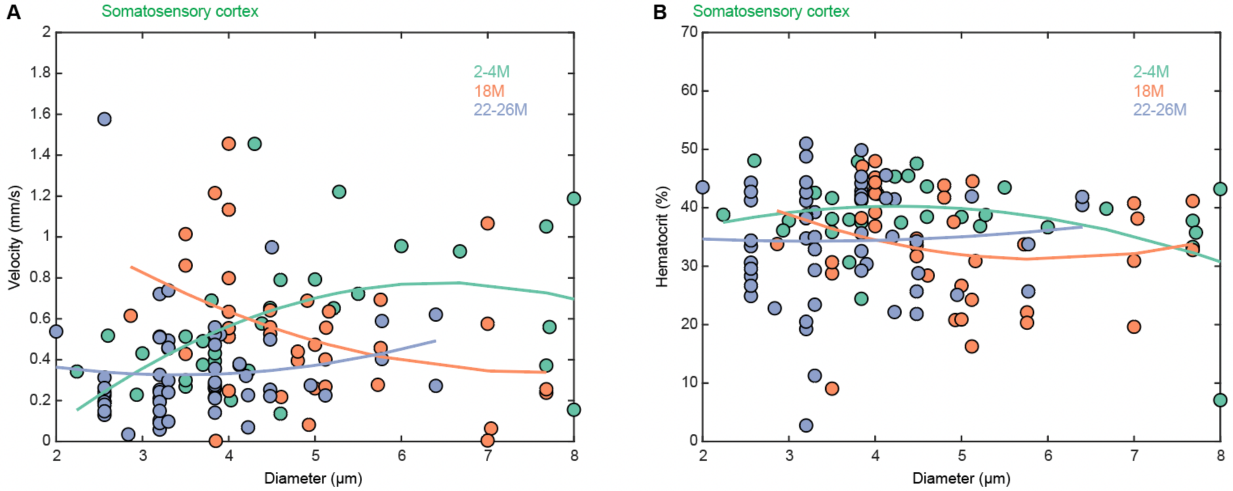

Red blood cell velocity and hematocrit in the capillary network. (**A**) Scatter plots showing basal RBC velocity as a function of capillary lumen diameter. Solid lines indicate a least mean square fit for all the data points in each group for 2-month: 32 capillaries, n = 10 mice (6 male and 4 female) and 18-month: 36 capillaries, n = 5 (all male) mice. (**B**) As in (**A**) but for instantaneous hematocrit for 2-month: n = 10 mice (6 male and 4 female) and 18-month: n = 5 (all male) mice.

Supplementary Text 1. Red blood cells spacing in the capillary network does not change with aging.

We quantified the spacing of RBC and compared the occurrence of "stall" events during different aging groups. Approximately 207 minutes data from 68 capillaries in 13 mice were analyzed. In young (2-4 month old) mice, for all the RBC intervals during long resting periods (approximately 104 minutes data from 32 capillaries in 8 mice; 29.4 ± 18.6 ms, median ± interquartile range; 95% confidence interval: [16.6 ms, 122.2 ms]), only approximately 0.02% RBC intervals are stall events (1.41 ± 1.37 second, median ± interquartile range; 95% confidence interval: [1.01 second, 6.56 seconds]). In 18-month old mice, for all the RBC intervals during long resting periods (approximately 103 minutes data from 36 capillaries in 5 mice; 31.4 ± 23.7 ms, median ± interquartile range; 95% confidence interval: [16 ms, 107.3 ms]), approximately 0.02% RBC intervals are stall events (1.77 ± 1.71 second, median ± interquartile range; 95% confidence interval: [1.00 second, 12.6 seconds]). These results suggest that there are no significant changes in stall occurrence in healthy aging, although mouse models of Alzhemier's disease show significant more occurrence of stall events.
