# Supplementary material for: Aging drives cerebrovascular network remodeling and functional changes in the mouse brain
Source: Nat Commun. 2024 Jul 30;15:6398. doi: 10.1038/s41467-024-50559-8 (PMC11289283; doi:10.1038/s41467-024-50559-8)
Supplement: Supplementary file 3 — Reporting Summary [file 41467_2024_50559_MOESM3_ESM.pdf]

nature portfolio

Corresponding author(s): Yongsoo Kim

Last updated by author(s): Jun 22, 2024

# Reporting Summary

Nature Portfolio wishes to improve the reproducibility of the work that we publish. This form provides structure for consistency and transparency in reporting. For further information on Nature Portfolio policies, see our Editorial Policies and the Editorial Policy Checklist.

## Statistics

For all statistical analyses, confirm that the following items are present in the figure legend, table legend, main text, or Methods section.

| n/a | Confirmed | |
|---|---|---|
| ☐ | ☒ | The exact sample size (*n*) for each experimental group/condition, given as a discrete number and unit of measurement |
| ☐ | ☒ | A statement on whether measurements were taken from distinct samples or whether the same sample was measured repeatedly |
| ☐ | ☒ | The statistical test(s) used AND whether they are one- or two-sided<br>*Only common tests should be described solely by name; describe more complex techniques in the Methods section.* |
| ☐ | ☒ | A description of all covariates tested |
| ☐ | ☒ | A description of any assumptions or corrections, such as tests of normality and adjustment for multiple comparisons |
| ☐ | ☒ | A full description of the statistical parameters including central tendency (e.g. means) or other basic estimates (e.g. regression coefficient) AND variation (e.g. standard deviation) or associated estimates of uncertainty (e.g. confidence intervals) |
| ☐ | ☒ | For null hypothesis testing, the test statistic (e.g. *F*, *t*, *r*) with confidence intervals, effect sizes, degrees of freedom and *P* value noted<br>*Give P values as exact values whenever suitable.* |
| ☒ | ☐ | For Bayesian analysis, information on the choice of priors and Markov chain Monte Carlo settings |
| ☒ | ☐ | For hierarchical and complex designs, identification of the appropriate level for tests and full reporting of outcomes |
| ☒ | ☐ | Estimates of effect sizes (e.g. Cohen's *d*, Pearson's *r*), indicating how they were calculated |

*Our web collection on statistics for biologists contains articles on many of the points above.*

## Software and code

Policy information about availability of computer code

| | |
|---|---|
| Data collection | STPT datasets were collected using acquisition computer and commercial software provided by TissueCyte.<br>LSFM datasets were collected using acquisition computer and commercial software provided by LifeCanvas.<br>In vivo imaging datasets using wide-field intrinsic optical imaging method were collected using custom code written in LabVIEW.<br>In vivo imaging datasets using two-photon laser scanning microscopy were collected using MScan (Sutter Instrument) and custom code written in LabVIEW. |
| Data analysis | Stitching of STPT and LSFM datasets and their analyses were done using custom built codes which were indicated in "Code Availability" section.<br>In vivo imaging datasets were analyzed using custom code written in Matlab. |

For manuscripts utilizing custom algorithms or software that are central to the research but not yet described in published literature, software must be made available to editors and reviewers. We strongly encourage code deposition in a community repository (e.g. GitHub). See the Nature Portfolio guidelines for submitting code & software for further information.

# Data

Policy information about availability of data

All manuscripts must include a data availability statement. This statement should provide the following information, where applicable:

- Accession codes, unique identifiers, or web links for publicly available datasets
- A description of any restrictions on data availability
- For clinical datasets or third party data, please ensure that the statement adheres to our policy

All datasets can be used for non-profit research without any restriction. Any additional information required to reanalyze the data reported in this paper is available from the lead contact upon request.

# Research involving human participants, their data, or biological material

Policy information about studies with human participants or human data. See also policy information about sex, gender (identity/presentation), and sexual orientation and race, ethnicity and racism.

| | |
|---|---|
| Reporting on sex and gender | *Use the terms sex (biological attribute) and gender (shaped by social and cultural circumstances) carefully in order to avoid confusing both terms. Indicate if findings apply to only one sex or gender; describe whether sex and gender were considered in study design; whether sex and/or gender was determined based on self-reporting or assigned and methods used. Provide in the source data disaggregated sex and gender data, where this information has been collected, and if consent has been obtained for sharing of individual-level data; provide overall numbers in this Reporting Summary. Please state if this information has not been collected. Report sex- and gender-based analyses where performed, justify reasons for lack of sex- and gender-based analysis.* |
| Reporting on race, ethnicity, or other socially relevant groupings | *Please specify the socially constructed or socially relevant categorization variable(s) used in your manuscript and explain why they were used. Please note that such variables should not be used as proxies for other socially constructed/relevant variables (for example, race or ethnicity should not be used as a proxy for socioeconomic status). Provide clear definitions of the relevant terms used, how they were provided (by the participants/respondents, the researchers, or third parties), and the method(s) used to classify people into the different categories (e.g. self-report, census or administrative data, social media data, etc.) Please provide details about how you controlled for confounding variables in your analyses.* |
| Population characteristics | *Describe the covariate-relevant population characteristics of the human research participants (e.g. age, genotypic information, past and current diagnosis and treatment categories). If you filled out the behavioural & social sciences study design questions and have nothing to add here, write "See above."* |
| Recruitment | *Describe how participants were recruited. Outline any potential self-selection bias or other biases that may be present and how these are likely to impact results.* |
| Ethics oversight | *Identify the organization(s) that approved the study protocol.* |

Note that full information on the approval of the study protocol must also be provided in the manuscript.

# Field-specific reporting

Please select the one below that is the best fit for your research. If you are not sure, read the appropriate sections before making your selection.

☒ Life sciences ☐ Behavioural & social sciences ☐ Ecological, evolutionary & environmental sciences

For a reference copy of the document with all sections, see nature.com/documents/nr-reporting-summary-flat.pdf

# Life sciences study design

All studies must disclose on these points even when the disclosure is negative.

| | |
|---|---|
| Sample size | For our vascular analysis, we used n=4 or 5 without sample size calculation. The chosen number is based on recent studies to map distribution of the vasculature in the mouse brain (Ji et al., 2021, Neuron; Kirst et al., 2020, Cell; Wu et al., 2022 Cell Reports). For the pericyte mapping, we use n = 10 based on our recent cell type mapping studies (Kim et al., 2017, Cell; Newmaster et al., 2020, Nature Communications; Wu et al., 2022 Cell Reports). For in vivo functional brain imaging, sample sizes were chose to be consistent with previous studies (Winder et al., 2017, Nature Neuroscience; Zhang et al., 2019, Nature Communications). |
| Data exclusions | For the vasculature analysis, we excluded dataset that did not pass our quality control step due to impartial vascular labeling. For PDGFRb-Cre:Ai14 for pericyte labeling, we exclude dataset with leaky neuronal labeling. For in vivo functional brain imaging involving whisker stimulation, auditory and ipsilateral stimuli were administered but omitted from the primary analysis because their responses were primarily related to stimulus-evoked movement (see our previous publication: Winder et al., 2017, Nature Neuroscience). |

| | |
|---|---|
| Replication | All our measurements were done once for the study. However, we carefully compared our brain volume, vascular and cell density measurements with published results in selected brain areas, confirming that our results are closely matched with previous measurements. For in vivo functional brain imaging, data were collected from the same animal for at least three different days. All animals in each age group showed similar hemodynamic response dynamics. |
| Randomization | This work does not contain experiments requiring randomization to test impact of experimental parameters between control and experimental groups. |
| Blinding | This work does not contain experiments requiring blinding. All vascular analysis and cell counting were performed by computer algorithms without any human bias. |

# Reporting for specific materials, systems and methods

We require information from authors about some types of materials, experimental systems and methods used in many studies. Here, indicate whether each material, system or method listed is relevant to your study. If you are not sure if a list item applies to your research, read the appropriate section before selecting a response.

## Materials & experimental systems

| n/a | Involved in the study |
|---|---|
| ☐ | ☒ Antibodies |
| ☒ | ☐ Eukaryotic cell lines |
| ☒ | ☐ Palaeontology and archaeology |
| ☐ | ☒ Animals and other organisms |
| ☒ | ☐ Clinical data |
| ☒ | ☐ Dual use research of concern |
| ☒ | ☐ Plants |

## Methods

| n/a | Involved in the study |
|---|---|
| ☒ | ☐ ChIP-seq |
| ☒ | ☐ Flow cytometry |
| ☒ | ☐ MRI-based neuroimaging |

## Antibodies

| | |
|---|---|
| Antibodies used | smooth muscle actin (Acta2): Rabbit anti-Acta2, Abcam, cat: ab5694<br>transgelin (Sm22): Rabbit anti-Sm22 Abcam, cat: ab14106<br>PDGFRGoat anti- PDGFR, R&D Systems, cat. no.: AF1042<br>Mouse Aminopeptidase N/CD13: Goat anti-CD13, R&D Systems, cat. no.: AF2335<br>Alexa Fluor® 488-AffiniPure Fab Fragment Donkey Anti-Rabbit IgG (H+L): Jackson ImmunoResearch laboratories, cat. no.: 711-547-003<br>Alexa Fluor® 647-AffiniPure Fab Fragment Donkey Anti-Goat IgG (H+L): Jackson ImmunoResearch laboratories, cat. no.: 705-607-003<br>Polyclonal rabbit anti-zo-1: Invitrogen Cat# 40-2200, RRID:AB_2533456<br>Donkey anti-rabbit conjugated with Alexa 568: Thermo Fisher Scientific Cat# A10042<br>anti-IgG: CF640R, Biotium Cat# 20177, RRID:AB_10853475 |
| Validation | We performed no primary antibody control experiment and confirmed lack of non-specific labeling. |

## Animals and other research organisms

Policy information about studies involving animals; ARRIVE guidelines recommended for reporting animal research, and Sex and Gender in Research

| | |
|---|---|
| Laboratory animals | All info related to the animal used in this study were included in "Animals" section in the Material and Methods |
| Wild animals | N/A |
| Reporting on sex | For in vivo functional brain imaging, both male and female mice were used between the ages of 2-18 months. |
| Field-collected samples | N/A |
| Ethics oversight | Animal experiments were approved by the Institutional Animal Care and Use Committee at Penn State University and Cold Spring Harbor Laboratory. |

Note that full information on the approval of the study protocol must also be provided in the manuscript.

## Plants

| | |
|---|---|
| Seed stocks | *Report on the source of all seed stocks or other plant material used. If applicable, state the seed stock centre and catalogue number. If plant specimens were collected from the field, describe the collection location, date and sampling procedures.* |
| Novel plant genotypes | *Describe the methods by which all novel plant genotypes were produced. This includes those generated by transgenic approaches, gene editing, chemical/radiation-based mutagenesis and hybridization. For transgenic lines, describe the transformation method, the number of independent lines analyzed and the generation upon which experiments were performed. For gene-edited lines, describe the editor used, the endogenous sequence targeted for editing, the targeting guide RNA sequence (if applicable) and how the editor was applied.* |
| Authentication | *Describe any authentication procedures for each seed stock used or novel genotype generated. Describe any experiments used to assess the effect of a mutation and, where applicable, how potential secondary effects (e.g. second site T-DNA insertions, mosiacism, off-target gene editing) were examined.* |
